# A replacement for islet equivalents with improved reliability and validity

**DOI:** 10.1007/s00592-012-0375-4

**Published:** 2012-02-03

**Authors:** Han-Hung Huang, Karthik Ramachandran, Lisa Stehno-Bittel

**Affiliations:** 1 Department of Physical Therapy and Rehabilitation Science, University of Kansas Medical Center, MS 2002, 3901 Rainbow Blvd., Kansas City, KS 66160 USA; 2 Bioengineering Graduate Program, University of Kansas, School of Engineering, Lawrence, KS 66045 USA

**Keywords:** Islet equivalent, Transplantation, Glut2, Proinsulin, Islet volume

## Abstract

Islet equivalent (IE), the standard estimate of isolated islet volume, is an essential measure to determine the amount of transplanted islet tissue in the clinic and is used in research laboratories to normalize results, yet it is based on the false assumption that all islets are spherical. Here, we developed and tested a new easy-to-use method to quantify islet volume with greater accuracy. Isolated rat islets were dissociated into single cells, and the total cell number per islet was determined by using computer-assisted cytometry. Based on the cell number per islet, we created a regression model to convert islet diameter to cell number with a high *R* ^2^ value (0.8) and good validity and reliability with the same model applicable to young and old rats and males or females. Conventional IE measurements overestimated the tissue volume of islets. To compare results obtained using IE or our new method, we compared Glut2 protein levels determined by Western Blot and proinsulin content via ELISA between small (diameter ≤ 100 μm) and large (diameter ≥ 200 μm) islets. When normalized by IE, large islets showed significantly lower Glut2 level and proinsulin content. However, when normalized by cell number, large and small islets had no difference in Glut2 levels, but large islets contained more proinsulin. In conclusion, normalizing islet volume by IE overestimated the tissue volume, which may lead to erroneous results. Normalizing by cell number is a more accurate method to quantify tissue amounts used in islet transplantation and research.

## Introduction

Islets of Langerhans are spherical-like clusters of endocrine cells that have a large range of sizes from 20 μm to more than 400 μm in diameter both in rodents [1] and humans [2, 3]. An accurate and consistent method to quantify the amount of tissue being used in experiments is of crucial relevance for islet research and transplantation. Prior to 1990 when Ricordi proposed islet equivalent (IE) calculations at the Second Congress of the International Pancreas and Islet Transplantation Association, results obtained between laboratories were incomparable [4]. According to IE calculations, one IE corresponds to the tissue volume of a perfectly spherical islet with a diameter of 150 μm. In the standard measurement procedure, a sample islet preparation is stained with dithizone (diphenylthiocarbazone) to discriminate islets from exocrine tissue. Under light microscopy, the diameter of individual islets is directly measured manually by the operator. Next, islets are categorized according to their diameters within 50-μm increments, and the number of islets in each category is multiplied by a related factor that converts the islet number and diameter category to IE [4, 5].

Currently, IE is the most common measurement used both in the clinic and in the laboratory. At transplantation sites, IE is a rapid measurement for quantifying the dosage of the transplanted material. The IE is used to estimate the yield of islets isolated from the donor, and the IE per kilogram of body weight is the unit commonly used to report the graft amount transplanted to the patient [6–22]. In the laboratory, IE is commonly applied to normalize the amount of islets between preparations for functional assays such as insulin secretion [1, 7, 23–25].

Recently, the accuracy, repeatability, and intermediate precision of the standard IE measurement procedure were tested in a multi-center study [26]. The results were very disappointing and even alarming. For example, more than 50% of centers overestimated the IE count on the same (photographic) samples compared to the expert standard. The intra-technician coefficients of variation (CVs) from one repeat count in the 35 technicians participating, which were calculated to assess the repeatability, ranged from 0 to a maximum of 42.5%, and approximately 30% of technicians had a CV% over 10%. In addition, the inter-technician CVs within each center were used to assess the intermediate precision, and the average inter-technician CV% was around 15%. Overall, these results indicated that the validity and reliability of IE measurements were unsatisfying.

In fact, the accuracy of the IE measurement has been challenged for years [2, 26–31], because the IE calculations are based on the assumption that all islets are spherical, an assumption that has already been suggested to be incorrect [3, 27, 30, 32–35]. In reality, most islets are disk-shaped oblate ellipsoid or irregular shaped, especially in culture. A measurement of the three largest dimensions in mutually perpendicular directions of the isolated islets has been reported. In a perfect sphere, the three major radii a, b, and c would equate to a = b = c. However, the average measured ratio of b/a was 0.82 and c/a was 0.6 suggesting that islets are more ellipsoidal [36].

Several digital image analysis methods have been proposed to replace manual estimation to improve quality assurance of islet products for transplantation [26, 27, 33, 35, 37–41]. By photographing islets digitally, the area and diameter of each islet were measured by computer and the islet volume (IE) was calculated accordingly. In addition, Buchwald, et al. [2] recently proposed a refinement of current IE measurement to improve the accuracy. However, these efforts were still based on the assumption that an islet is a sphere. Therefore, we suggest that either the one-dimensional (longitudinal axis) or two-dimensional (area) approach of IE measurement used currently is an oversimplification, leading to an inaccuracy when estimating the actual tissue amount of a three-dimensional islet.

In the present study, we developed and tested a new method for estimating islet volume based on cell number counts. We completed validity and reliability studies on our new method and showed that it is a fast and more accurate and reliable method of estimating islet volume. We are freely distributing worksheets embedded with the new conversion calculations to assist all laboratories and clinics working with isolated islets. These efforts are relevant for improving the accuracy of transplanted tissue volume and assuring that erroneous results are not reported in the literature.

## Methods

### Rat islet isolation, separation and IE measurement

Adult male and female Sprague–Dawley rats (200–350 g BW) were housed on a 12 h light/dark cycle with free access to standard laboratory chow and water. All animals received care in compliance with the Principles of Laboratory Animal Care formulated by the National Society for Medical Research and the Guide for the Care and Use of Laboratory Animals published by the US National Institutes of Health (NIH Publication No. 85–23, revised 1996).

Islet isolation methods followed our published procedures described in detail [1, 24, 25, 42]. Briefly, rats were anesthetized by intraperitoneal injection of a mixture of ketamine and xylazine. After the peritoneal cavity was exposed, the pancreatic main duct to the intestine was clamped and the pancreas cannulated in situ via the common bile duct. The pancreas was distended with collagenase and removed. Islets were gently tumbled, washed, and passed through a sterile 30 mesh stainless steel screen and centrifuged. The pellet was mixed with Histopaque and centrifuged, and the islets floating on the gradient were collected and sedimented. Islets were passed through a sterile 40-μm mesh cell strainer with HBSS. After this cleaning process, islets were placed into CMRL1066-based or DMEM/F12-based culture medium and put into a 37°C culture chamber containing 5% CO_2_. For manual separation of islets, the islet culture media was changed to L15 containing 10% FBS and 5 mM HEPES, and islets were transferred into 37°C culture chamber without CO_2_. After measuring the IE, the selected islets were frozen by liquid nitrogen and preserved in −80°C for subsequent protein and DNA analysis.

The IE measurements for each preparation followed our previously published procedures [1, 24, 25]. Briefly, the diameter of each islet was recorded manually using light microscopy at 40× total magnification. For irregularly shaped islets, two to four diameter measurements were taken at different locations on the islet and averaged for the final diameter measurement. The volume of each islet was calculated based on the diameter and converted to IE individually, where one IE is equal to 1.77 × 10^6^ μm^3^ (the volume of a spherical islet with 150 μm diameter) [2, 23, 27, 29, 38].

### Islet dissociation and cell number estimation

For single cell assays, isolated islets were picked manually and individually distributed into 96-well plates in medium containing calcium/magnesium-free HBSS. After recording the diameter of each islet as described above, the islets were dissociated into single cells using our published protocol [24, 42]. Briefly, after adding papain into each well with a final concentration of 5 U/ml, islets were incubated at 37°C for 20 min. Following that, the islets were dispersed into single cells by repeated pipetting, and the dissociated cells in the wells were spun down in the plate with 300 rpm for 1 min at room temperature. The cell number in each well was analyzed using Celigo™ adherent cell cytometer (Cyntellect Inc.). Every well containing single cells was photographed digitally, and the cells were counted using the Celigo software (1.3). Cells were counted from at least 340 islets from both male and female rats of two different age classifications (2 months old or 6 months old).

To test the reliability of the cell count, manual counts using a hemocytometer were performed. Islets were grouped manually into size categories based on diameters. The number of islets in each group was recorded before dissociating the islets into single cells based on our published protocol. A 10 μl sample of dispersed cells was loaded into a hemocytometer for cell counting. Six repetitions were performed in each sample set.

### Total protein yield

Islets within groups were homogenized using a 26^1^/_2_ syringe with extraction buffer containing 10 mM TRIS HCl pH7.4, 150 mM NaCl, 1 mM EDTA, 20 mM Na Molybolate, 50 mM Na Fluoride, 0.2 mM Na-Orthovanidate (pH 10), 1% Triton X-100, and 0.2 mM PMSF. The extracts were centrifuged for 15 min with 15,600 rcf at 4°C. Measurement of protein concentrations in supernatants was performed using Micro BCA Protein Assay Kit (Pierce, #23235).

### Total DNA yield

Islets were lysed with lysis buffer containing 10 mM Tris–HCl pH 7.5, 150 mM NaCl, 5 mM EDTA (pH 8.0), 0.5% SDS, and 50 μg/ml proteinase K, and vortexed until the cell pellet was dispersed. After incubation overnight at 55°C, the sample was spun at 12,000 rpm for 10 min at room temperature. The supernatant was collected, and equal volume of isopropanol was added into each sample. After a 5 min rest, another centrifugation (12,000 rpm for 10 min at RT) was performed. The supernatant was discarded, and TE (Tris–EDTA) buffer was added to dissolve the DNA with a 1-h incubation at 55°C. The DNA concentrations were measured using Quant-iT™ PicoGreen^®^ dsDNA Assay Kit (Invitrogen, #P11496).

### Western Blot

Hand-picked small (diameter ≤ 100 μm) and large islets (diameter ≥ 200 μm) were washed individually in phosphate-buffered saline (PBS) twice. After removing the supernatant, the islets were homogenized as described previously. Samples were prepared for electrophoresis by heating at 95°C for 3 min in SDS gel-loading buffer (0.125 M Tris, pH 6.8, 5% glycerol, 2.5% mercaptoethanol, 2% SDS, and 0,001% bromophenol blue). Proteins were separated on a 4–15% Tris–HCl Ready Gels (Bio-Rad Laboratories, #161–1158) with 0.025 M Tris, 0.192 M Glycine, 0.1% SDS running buffer. Equal amounts of total protein (10 μg) were loaded in each lane. Molecular weight markers See Blue Plus2 Pre-Stained Standard (Invitrogen, #LC5925) was used to determine the size of the antigen. After electrophoresis, the proteins were transferred from the gel to Bio Trace PVDF membranes 0.45 μm (Pall Life Sciences, #P/N 66547) using 0.012 M Tris, 0.096 M Glycine transfer buffer. Blots were blocked with 5% nonfat dry milk diluted in 0.1 M PBS 0.1% Tween (PBST) for 1 h. Primary and secondary antibodies were diluted in the 5% nonfat dry milk in PBST. All incubations were performed at room temperature. Blots were probed with primary antibodies against Glut2 (Santa Cruz Biotechnology Inc., #sc-9917), for 1 h at room temperature. After washing in 0.1 M PBS 0.1% Tween (10 min for 3 times), blots were incubated for 30 min with secondary antibody horseradish peroxidase-conjugated goat anti-rabbit IgG (Santa Cruz Biotechnology Inc., #sc-2004) or goat anti-mouse IgG (Santa Cruz Biotechnology, #sc-2005). After washing in 0.1 M PBS 0.1% Tween (10 min for 3 times), bound antibodies were detected using SuperSignal^®^ West Pico Chemiluminescent Substrate (Thermo Fisher Scientific Inc., # 34080). For a protein loading control, the membrane was reprobed with mouse anti-GAPDH (Sigma-Aldrich^®^, #G8795), for 1 h at room temperature.

### Proinsulin content

Isolated islets were placed in 24-well plate with a minimum of 5 large or 15 small islets per well. All wells were preincubated for 2.5 h in RPMI 1640 containing 10% fetal bovine serum and 3 mM glucose in a 37°C containing 5% CO_2_. After preincubation, media was removed and fresh media added. After a 30-min static incubation at 37°C and 5% CO_2_, the islets were harvested and frozen at −80°C. The total protein in the islets was extracted by sonication in acid ethanol (0.18 M HCl in 95% ethanol) and incubated overnight at 4°C. The total intracellular proinsulin amounts were determined by the ELISA (ALPCO, # 80-PINRT-E01).

### Statistics

The exact number of islets or replicates is shown in each figure legend. Results were expressed as means of each group or cell population ± SEM and were compared using the Student’s *t* test. The Pearson product-moment correlation was used to test the correlation between the two cell counting techniques. When comparing the regression equations of young and adult and male and females rat islets, analysis of covariance (ANCOVA) was used. Significant differences were defined as *p* < 0.05.

## Results

### Cell number per islet

The dissociated islet cells were counted using computer-assisted cytometry. The total cell number per islet from different sizes of islets is summarized in Table 1. There was an average of 943 cells in a 150 μm-diameter islet. Based on the measured cell numbers, a third-order polynominal regression trend line was the best fit with the equation:

$$ y = - 0.0001x^{3} + 0.0912x^{2} - 6.2162x + 182.1125 $$

where *y* equals the total cell number and *x* equals the islet diameter (μm).

**Table 1:** Cell number per islet estimated by computer-assisted cytometer

Islet diameter (μm)	Number of islets evaluated^a^	Cell number per islet
50	25	92 ± 11
75	48	188 ± 15
100	40	322 ± 24
125	36	642 ± 48
150	33	943 ± 60
175	46	1,308 ± 68
200	42	1,674 ± 91
225	31	2,099 ± 94
250	21	2,731 ± 137
275	7	2,831 ± 216
300	5	3,586 ± 689
325	9	4,003 ± 506

The mean cell number per islets of various size categories is shown. Data are presented in mean ± SEM

^a^The islets were harvested from 8 animals

**Fig. 1:**
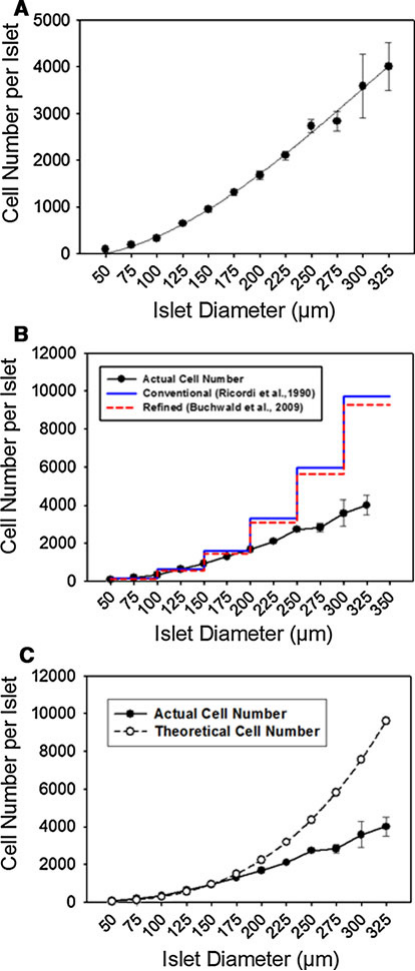
IE measurements overestimated the actual tissue volume in large islets. **a** The counted cell number per islets was plotted, and a third-order polynomial regression trend line with a *r* ^2^ of 0.8 produced the best fit with the data. **b** The measured relationship between islet size and actual cell number per islet was plotted in the *black solid line*. Two theoretical curves were plotted using the Ricordi’s conventional IE measurement with different categories of sizes in a 50-μm increments (*blue* step plot) [4] and using Buchwald’s refined IE measurement (*red* step plot) [2], based on our measured 943 cells per 150-μm diameter islet (1 IE). **c** Theoretical curve (*dash line*) was plotted based on the assumptions that islets are perfect spheres and all cell sizes are equivalent (color figure online)

The *R* ^2^ value was 0.8, indicating the regression trend line fit the data well (Fig. 1a).

Next, we compared our measured cell number per islet with the conventional IE measurement by Ricordi et al. [4] and the refined IE measurement by Buchwald et al. [2]. To make the calculation, we used our measured 943 cells per an average 150-μm diameter islet for the Ricordi and Buchwald calculations. The results were plotted in Fig. 1b. Compared to the our actual cell counts, the difference between the actual and theoretical cell counts (Ricordi and Buchwald) was more prominent as the islets increased in diameter, especially in islets over 200 μm in size. Even though Buchwald’s refined algorithm introduced a downward correction, the adjustment was marginal and a significant overestimation still existed (Fig. 1b). In addition, we compared a theoretical curve with the assumption that all islets are perfect spheres. As shown in Fig. 1c, this sphere-based curve was also different from the actual cell count especially for large islets.

### Cell number per IE

**Fig. 2:**
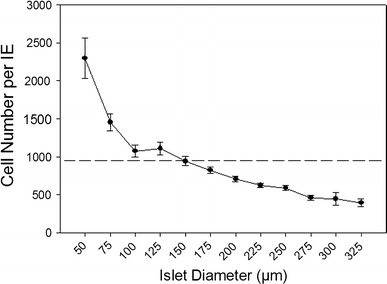
Cell number per IE. The flat* dashed line* represents 943 cells per IE based on the assumption that IE calculations accurately reflected the true volume of any sizes of islets. However, according to the results of actual cell count, there was a negative correlation between islet size and cell number per IE

To further demonstrate the errors within the current IE calculations, we plotted the cell number within islets divided by the islet’s IE based on the Ricordi method [4]. Figure 2 illustrates the errors within the current IE calculations. The dotted line indicates the cell number/IE that should be obtained independent of the size of the islet, if IE were an accurate measure of islet volume. However, our measured values (solid line) demonstrate that the IE calculation overestimated the true volume with increasing size, leading to possible erroneous results when IE is used as a normalization method.

### Validity

#### Total DNA content per IE and per cell

When total DNA per islet prep was normalized by IE, the results indicated that small islets had a significantly higher DNA content per volume compared to large islets (*p* < 0.001). The total DNA content, when calculated using our cellular regression, showed no difference (*p* = 0.43) in small and large islets (Table 2). Using our cell number normalization, the average DNA content per cell was approximately 6.05 ± 0.69 pg, which is in agreement with other reports of total DNA/cell [30, 43, 44].

**Table 2:** Total DNA content per IE and per cell in small and large islets

	Small islets	Large islets	*p* value
DNA (pg)/IE	8.19 ± 1.12	3.62 ± 0.70	<0.001
DNA (pg)/cell	6.17 ± 0.97	5.93 ± 1.00	0.43

DNA levels were measured from groups of large and small islets and normalized to volume using standard IE calculations (upper row), and normalized to cell number (bottom row). When the two groups of islets were compared after IE normalization, there was statistically more DNA in the small islets/volume. However, when normalized to cell number, the DNA content did not differ between islets groups. For small islets,* n* = 21 experiments from 6 rats. For large islets,* n* = 20 experiments from 6 rats. Each experiment contained at least 50 small islets and 20 large islets

#### Total protein content per IE and per cell

When total protein within an islet was normalization to IE, the result indicated that small islets contained a significantly higher total protein amount than large islets (*p* < 0.05, Table 3). However, when the same raw data were normalized using our cellular regression, there was no significant difference (*p* = 0.35) in protein content per cell between small and large islets. In addition, total protein content per DNA content was calculated, since DNA content is commonly used for cell number normalization. No difference (*p* = 0.40) was noted in total protein per DNA between small and large islets (Table 4), further validating our method of islet volume normalization.

**Table 3:** Total protein content per IE and cell in small and large islets

	Small islets	Large islets	*p* value
Protein (μg)/IE	0.82 ± 0.07	0.50 ± 0.08	<0.05
Protein (ng)/cell	0.58 ± 0.06	0.69 ± 0.10	0.35

Total protein levels were measured from groups of large and small islets, normalized to volume using standard IE calculations (upper row), and normalized to cell number (bottom row). When the two groups of islets were compared after IE normalization, there was statistically more protein in the small islets/volume. However, when normalized to cell number, the total protein level did not differ between islets groups. Each experiment contained a minimum of 50 large islets and 450 small islets. *n* = 4 rats for each group

**Table 4:** Total protein content per DNA in small and large islets

	Small islets	Large islets	*p* value
Protein (μg)/DNA (ng)	0.13 ± 0.00	0.12 ± 0.01	0.40

To further validate the use of cell number as a normalizing factor, the protein/cell and DNA/cell were divided. The values were not different between the two islet size groups, and compared favorably to previously published data.* n* = 4 experiments from 2 rats. Each experiment contained at least 250 small islets or 20 large islets

### Reliability

**Fig. 3:**
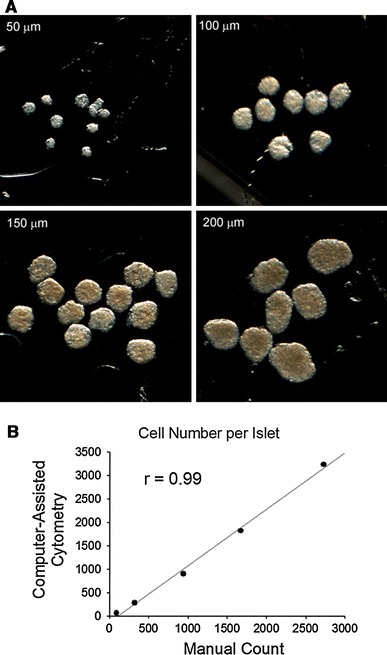
The isolated rat islets. **a** The isolated islets with high purity were manually separated into specific size categories (diameter of 50, 100, 150, 200, and 250 μm). The images illustrate the low variability in size within category. **b** The correlation between cell count using the computer-assisted cytometry and manual counting methods shows that the two had excellent agreement. Each sample contained more than 256 islets in 50 μm, 94 islets in 100 μm, 83 islets in 150 μm, 37 islets in 200 μm, or 16 islets in 250 μm from 2 adult male rats. The data from manual counting method were correlated with the data shown in Table 1

To test inter-method reliability, islets were manually separated into five categories based on diameters of 50, 100, 150, 200, and 250 μm. Figure 3a provides examples of the uniform separation that was achieved for each size category. After dissociation, the cell number per islet in each category was evaluated using a hemocytometer. The results showed a high correlation (*r* = 0.99) in cell counts per islet between the hemocytometer counts and the computer-assisted cytometer indicating good inter-method reliability (Fig. 3b).

**Fig. 4:**
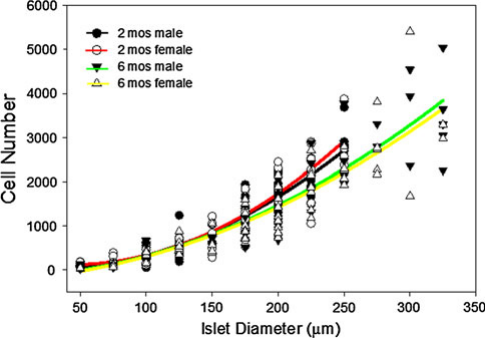
Age and gender did not alter the cell number method. Islets from 2-month-old male (*black line*) and female (*red line*) and 6-month-old male (*green line*) and female (*yellow line*) rats were separated into size groups and the cell number for each determined. There was no statistical difference in the regression lines through 250 μm diameters. *r* ^2^ value for each regression equation is: 2 months males *r* ^2^ = 0.86, 2 months females *r* ^2^ = 0.87, 6 months males *r* ^2^ = 0.87, 6 months females, *r* ^2^ = 0.78. *n* = 102 islets for 2-month-old males, 101 islets for 2-month-old females, 125 for 6-month-old males, and 121 for 6-month-old females (color figure online)

The applicability of the new method of islet volume normalization was tested on islets from young and adult as well as male and female rats. Islets were obtained from 2 months of female and male rats and 6-month-old female and male rats, and the cells per islet were counted. Figure 4 plots the relationship between islet diameter and cell number, showing that there was little difference in the regression curves between the four groups. Analysis of the covariance of the groups through islet diameters up to 250 μm indicated that there was no statistical difference between the groups. Larger islets were excluded from analysis because the 2-month-old male and female rats did not have larger islets.

### Application of model: Glut2 protein levels

As an example of how IE measurements can alter the conclusions drawn from an experiment, Glut2 and proinsulin levels were measured from small and large islets and the data were normalized using the traditional IE [4, 5] and our cell-based method.

**Fig. 5:**
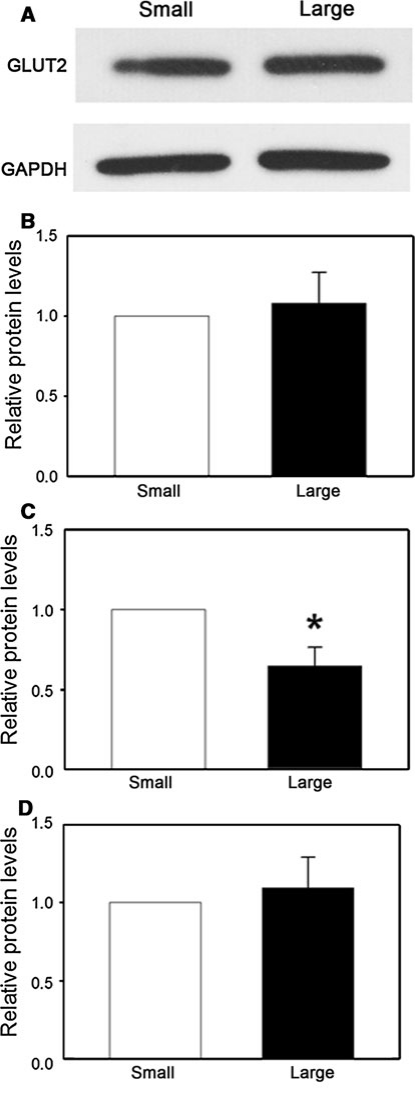
Glut2 protein levels in small and large islets. **a** Bands from a representative Western Blot probing for GLUT2 are shown. **b** The blot densitometry data were normalized to the internal control GAPDH; **c** Normalization to GAPDH and IE; **d** Normalization to GAPDH and cell number. The data are presented as the relative fold differences to small islets. *n* = 5 experiments, each experiment contained at least 1,500 small islets or 300 large islets from 6 adult male rats. (*p < 0.05)

A representative Western Blot of Glut2 is shown in Fig. 5a. When the Western Blot densitometry was normalized to the levels of the internal control, GAPDH, there was no significant difference (*p* = 0.69) in the Glut2 protein levels between small and large islets (Fig. 5b). IE values and the total cell number resulting in 10 μg of protein in large and small islets were calculated based on the results shown in Table 3. The number of cells required to isolate 10 μg of protein was not statistically different between large and small islets when normalized by cell number (*p* = 0.37) (Table 5). However, using the IE-based volume calculation, more IE of large islets was needed to isolate the same amount of protein compared to small islets (*p* < 0.05).

**Table 5:** IE and total cell number to obtain 10 μg of protein in small and large islets

	Small islets	Large islets	*p* value
IE/10 μg protein	12.43 ± 1.12	21.84 ± 3.35	<0.05
Cell/10 μg protein	17,855 ± 1,698	15,379 ± 1,910	0.37

To further validate the use of cell number as a normalizing factor, the volume of islets needed to obtain 10 μg of protein were analyzed using either IE or cell number. When the IE calculation was done, nearly twice the amount of large islet volume was needed to obtain 10 mg of protein, compared to small islets. When normalized by cell number, the values were not different between the two islet size groups. *n* = 4 samples. Each sample contained more than 450 small islets or 50 large islets from 1 rat

When the blot densitometry was normalized by IE, Glut2 levels were significantly higher (*p* < 0.05) in small islets than in large islets (Fig. 5c). However, when normalized by total cell number, there was no significant difference of Glut2 protein levels (*p* = 0.25) between the two groups (Fig. 5d).

### Application of model: proinsulin content

**Fig. 6:**
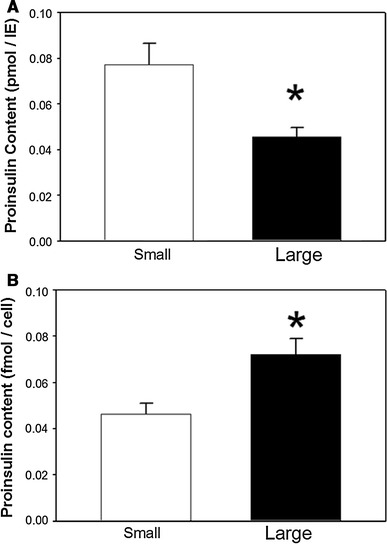
Proinsulin content in small and large islets. **a** When normalized by IE, small islets showed a higher proinsulin content; **b** When normalized by cell number, the results were opposite, showing that large islets had a higher proinsulin content. *n* = 3 experiments, each experiment contained two replicates with at least 16 small islets or 6 large islets per replicate from 2 adult male rats. (*p < 0.05)

Proinsulin content in small and large islets was determined by ELISA. The results also varied based on the normalization methods. When normalized by IE, the proinsulin levels were significantly lower (*p* < 0.05) in the large islets compared to the small islets (Fig. 6a). However, when normalized by the cell number, the results were opposite with significantly higher levels (*p* < 0.05) in the cells of the large islets compared to the small (Fig. 6b).

## Discussion

The accuracy of the international-standardized IE measurement has been questioned for years. Several factors that may affect the accuracy have been recognized, such as subjective judgment of the examiners, sampling techniques, and islet purity. While most research groups were engaged in studying computer-assisted digital image analysis (DIA) to compensate for these disadvantages, the accuracy of the algorithm that converts islet size into IE has received little attention until recently. Buchwald et al. suggested that the algorithm, first proposed by Ricordi et al. [2], overestimated total IEs by 4–8%. Accordingly, they proposed modified conversion factors to estimate IE. However, here we showed that both the conventional IE measurement and the Buchwald’s refined measurement overestimated the actual tissue volume in large islets. It has been suggested that grouping islets into a 50-μm range in current standard procedure might lead to a overestimation in IE [2, 27]. We suggest that the 50-μm categories along with the incorrect assumption that islets are spheres in the current algorithm causes IE measurement inaccuracy, because many research groups, in addition to us, have recognized that the shapes of islets are irregular [3, 27, 30, 32–35].

Since islets are cluster of cells, we proposed to estimate islet tissue volume by total cell number. Recently, Pisania et al. [31] estimated the islet volume by using cell nuclei count. They concluded an overestimation when using IE measurement and suggested the overestimation might be due to the possible space such as intra-islet vessel and intracellular space not accounted for by IE measurements. Compared to Pisania’s work, we also base our volume estimations on cell numbers, but our approach does not require an extra step in the experimental process (nuclei staining).

We developed a regression model to estimate the number of cells per islet over a wide range of islet diameters with a high *R* ^2^ value and a good validity and reliability. When comparing the standard IE measurement to our method using cell number, we identified a significant overestimation of tissue volume in large islets by using IE calculations. The inverse correlation between total cell number per IE and islet size can explain this finding, because when the total cell number was normalized to IE, there were fewer cells per IE in large islets compared to small islets, which might be due to more intracellular space and vessels in the large islets as described previously [31].

Current volume normalizing methods in islet research need to be reconsidered, because completely different results may be obtained depending to the normalization method. Here, we provide examples of Western Blot to study the Glut2 protein levels and ELISA to report proinsulin content comparing small and large islets. We show contradicting results based solely on the normalization method used. One could question which method is the most accurate. We show that normalization by cell number provides nearly identical amounts of total DNA/cell and total protein/cell. Normalization by IE indicates that there is statistically more DNA and total protein/IE in small islets compared to large (Tables 2 and 3). Since DNA and total protein per volume should be consistent values regardless of the size of the islet, the cell number normalization method is less likely to lead to erroneous results.

Our findings may have a tremendous impact on assumptions made about the volume versus size of islets within the rat pancreas. It is frequently reported that large islets (diameter > 150 μm) comprise 47% of total volume of endocrine tissue from the pancreas, even though they comprise a relatively small number (5% of the total number of islets) [45]. Moreover, when considering islets of 100 μm in diameter and greater, it is thought that they make up 20% of the total number of islets but nearly 75% of the total islet volume [45]. However, those estimates are based on Hellman’s early work that still considered islets as spherical [46]. Thus, the actual percentage of volume defined by large islets may be less and needs to be reconsidered.

In clinical transplant settings, the implications may be immense. Large islets are preferentially used for transplantation due to their relatively high assumed tissue volume. However, the volume of large islets has to be assessed more carefully, because recently the high variability in IE measurement due to large islets has been recognized by others [2]. We suggested that if one calculates the typical volume of islets transplanted during the process and corrects it based on Fig. 1a, then the actual volume transplanted tissue will be significantly less. However, in order to complete those assessments, a separate conversion model must be determined for human islets. Currently, we are in the process of completing those studies.

In order to freely distribute the method described here for researchers, we created a spreadsheet that automatically calculates cell number from any measured islet diameter between 20 and 350 μm. The spreadsheet is available online for free public downloads at http://www.ptrs.kumc.edu/kansasmethod/ or http://ptrs.kumc.edu/kansasmethod/. When the human conversion factor is published, we will provide a separate spreadsheet for automatic conversion of human islets to cell counts at the same URL. One of the major advantages of our new method is that it no longer relies on the technician’s ability to categorize an islet according to the conventional 50-μm classifications. Rather, it can be combined with digital programs that automatically calculate diameter, and an islet of any size, within a normal range, can be converted to cell number.

In conclusion, the assumption of spherical islets, which are the basis of the conventional IE measurements, is incorrect. IE measurements overestimate the islet volume, potentially affecting the results of islet research. We established a new method to estimate islet volume via the total cell number per isolated islet in rats. This model needs to be further established in humans to better estimate the tissue volume used in human islet research and transplantation.
